# Supplementary material for: Multi-Drug Resistant Staphylococcus aureus Carriage in Abattoir Workers in Busia, Kenya
Source: Antibiotics (Basel). 2022 Dec 1;11(12):1726. doi: 10.3390/antibiotics11121726 (PMC9774130; doi:10.3390/antibiotics11121726)
Supplement: Supplementary file 1 [file antibiotics-11-01726-s001.zip › antibiotics-2038424-supplementary.pdf]

Supplementary **Table S1**. Sequence types of MSSA and MRSA strains isolated from nasal of HIV positive and negative abattoir workers Busia County

**Sequence types**	**Total number n=126 (%)**	**Number isolated from HIV+ abattoir workers n=27 (%)**	**Number isolated from HIV-Abattoir workers n=99 (%)**
ST152	43 (34.1%)	8 (29.6%)	35 (35.4%)
ST8	19 (15.1%)	11 (40.7%)	8 (8.1%)
ST72	9 (7.1)		9 (9.1%)
ST80	7 (5.6%)		7 (7.1%)
ST22	5 (4.0%)	1 (3.7%)	4 (4.1%)
ST1	4 (3.2%)	1 (3.7%)	3 (3.1%)
ST25	4 (3.2%)	2 (7.4%)	2 (2.0%)
ST1633	4 (3.2%)		4 (4.0%)
MSSA ST88	4 (3.2%)	1 (3.7%)	3 (3.0%)
MRSA ST88	3 (2.4%)	1 (3.7%)	2 (2.0%)
ST1290	3 (2.4%)		3 (3.0%)
ST5	3 (2.4%)	1 (3.7%)	2 (2.0%)
ST707	2 (1.6%)		2 (2.0%)
ST30	3 (2.4%)	1 (3.7%)	2 (2.0%)
ST15	2 (1.6%)		2 (2.0%)
ST188	2 (1.6%)		2 (2.0%)
ST6	2 (1.6%)		2 (2.0%)
ST97	1 (0.8%)		1 (1.0%)
ST661	1 (0.8%)		1 (1.0%)
ST2430	1 (0.8%)		1 (1.0%)
ST101	1 (0.8%)		1 (1.0%)
ST1292	1 (0.8%)		1 (1.0%)
ST2126	1 (0.8%)		1 (1.0%)
ST573	1 (0.8%)		1 (1.0%)
Total	**126**	**27**	**99**
